# Clinical Validation of Automated Corrected QT-Interval Measurements From a Single Lead Electrocardiogram Using a Novel Smartwatch

**DOI:** 10.3389/fcvm.2022.906079

**Published:** 2022-06-23

**Authors:** Diego Mannhart, Elisa Hennings, Mirko Lischer, Claudius Vernier, Jeanne Du Fay de Lavallaz, Sven Knecht, Beat Schaer, Stefan Osswald, Michael Kühne, Christian Sticherling, Patrick Badertscher

**Affiliations:** ^1^Department of Cardiology, University Hospital Basel, Basel, Switzerland; ^2^Cardiovascular Research Institute Basel, University Hospital Basel, Basel, Switzerland

**Keywords:** QTc, smartwatch, intelligent ECG, digital health, artificial intelligence, remote patient monitoring (RPM), single-lead ECG

## Abstract

**Introduction:**

The Withings Scanwatch (Withings SA, Issy les Moulineaux, France) offers automated analysis of the QTc. We aimed to compare automated QTc-measurements using a single lead ECG of a novel smartwatch (Withings Scanwatch, SW-ECG) with manual-measured QTc from a nearly simultaneously recorded 12-lead ECG.

**Methods:**

We enrolled consecutive patients referred to a tertiary hospital for cardiac workup in a prospective, observational study. The QT-interval of the 12-lead ECG was manually interpreted by two blinded, independent cardiologists through the tangent-method. Bazett’s formula was used to calculate QTc. Results were compared using the Bland-Altman method.

**Results:**

A total of 317 patients (48% female, mean age 63 ± 17 years) were enrolled. HR-, QRS-, and QT-intervals were automatically calculated by the SW in 295 (93%), 249 (79%), and 177 patients (56%), respectively. Diagnostic accuracy of SW-ECG for detection of QTc-intervals ≥ 460 ms (women) and ≥ 440 ms (men) as quantified by the area under the curve was 0.91 and 0.89. The Bland-Altman analysis resulted in a bias of 6.6 ms [95% limit of agreement (LoA) –59 to 72 ms] comparing automated QTc-measurements (SW-ECG) with manual QTc-measurement (12-lead ECG). In 12 patients (6.9%) the difference between the two measurements was greater than the LoA.

**Conclusion:**

In this clinical validation of a direct-to-consumer smartwatch we found fair to good agreement between automated-SW-ECG QTc-measurements and manual 12-lead-QTc measurements. The SW-ECG was able to automatically calculate QTc-intervals in one half of all assessed patients. Our work shows, that the automated algorithm of the SW-ECG needs improvement to be useful in a clinical setting.

## Introduction

During the COVID-19 pandemic, the need for affordable and simple-to-use end-consumer solutions for health monitoring spiked (1). During the intake of certain medications, screening and monitoring for QT prolongation with frequent ECG checks is indicated and critical for patient safety. The Withings Scanwatch (SW, Withings Scanwatch, Withings SA, Issy les Moulineaux, France) offers automated analysis of the corrected QT-interval (QTc) remotely without the need for third-party software, manual measurement of SW-ECG or requiring different device positions during recording (2). Prior reports using other smart devices focused on the feasibility regarding manual measurements of the QT-interval *via* a single, six or more lead ECG (2–7). However, there is limited data available regarding the clinical validation of artificial intelligence (AI)-generated QT-measurements from commercially available smart devices (4, 5). We therefore sought to validate the use of automated SW-QTc-measurements in unselected patients referred to a cardiology service. The aim of this study was to compare automated QTc-measurements using a single lead ECG of a novel smart device compared to manual-measured QTc from a standard 12-lead ECG.

## Methods

We conducted a prospective, observational study enrolling consecutive adults (≥ 18 years) presenting to the University Hospital Basel from May 7 to June 18 2021 referred for obtaining a 12-lead ECG. The study was approved by the local ethics committee and complied with the Declaration of Helsinki. Written informed consent was provided by all participants. A trained nurse performed a 12-lead ECG immediately before or after instructing the patient in recording a single-lead ECG with the SW. These recordings were considered nearly simultaneously. To obtain a SW-ECG, patients were instructed to hold the stainless steel ring on the top case of the SW continuously for 30 s. Readings from the automated SW-algorithm (HR, PR-, and QT-interval plus QTc) were recorded and a PDF-file of the 30 s single-lead (lead I) SW-ECG was saved. QT-interval on the corresponding 12-lead ECG was manually interpreted by two blinded, independent cardiologists applying the tangent-method (8), using lead II or V5/V6 as suggested in previous work (6, 8, 9). Bazett’s formula was used to calculate QTc (10). If the discrepancy between the two manual 12-lead ECG measurements was < 20 ms, the mean of the two was taken to compare against the AI-determined measurement. If there was a mismatch ≥ 20 ms between the two QT-measurements in the 12-lead ECG, a third cardiologist performed a re-measurement. In this case, the mean of the two measurements with the least difference of the three QT-measurements was used to compare against the AI-measurement.

Agreement between QTc-measurements (manual QTc on 12-lead ECG and AI-measured SW-ECG) were assessed applying the Bland-Altman method. Mean difference (bias) in the QTc-interval between the two methods was calculated, so was the lower and upper limits of agreement (LoA, defined as ± 1.96 standard deviations). The same method was applied to show discrepancy between the manual measurements of the 12-lead ECGs by the cardiologists. The percentage of AI-SW and manual measurements that differed < 15, > 20, and > 30 ms for QTc have also been calculated. Area under the receiver operator characteristic curve (AUC) was used to determine the efficacy of the algorithm to discriminate measurements ≥ 460 ms in women and ≥ 440 ms in men.

## Results

**FIGURE 1:**
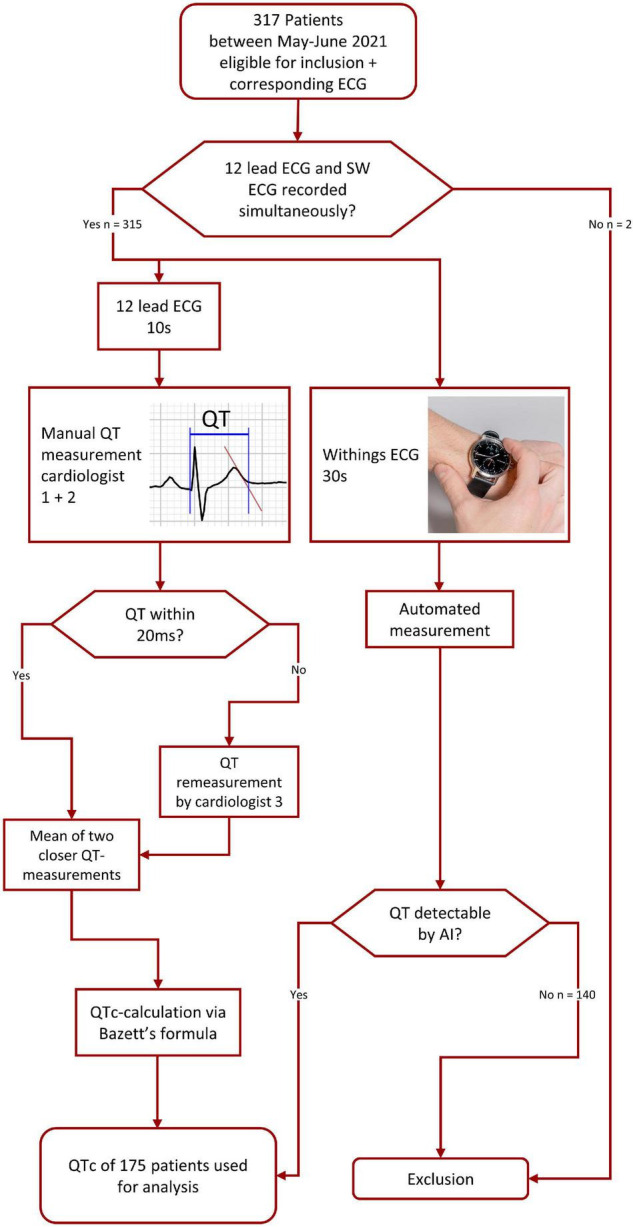
Flowchart showing pathway of pre-analysis data management and acquisition. AI, artificial intelligence.

**FIGURE 2:**
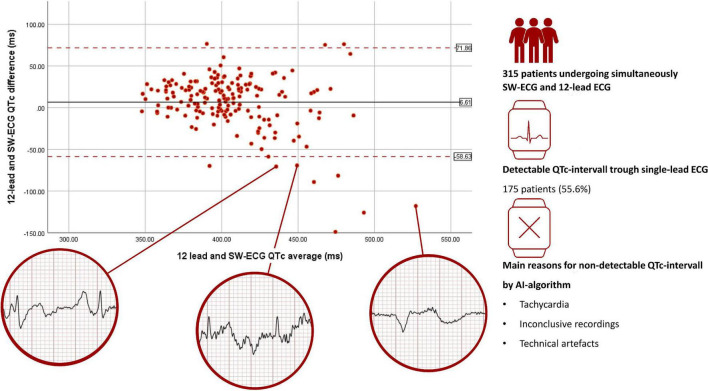
Comparison of QT-interval measurements, manually measured QTc on a 12-lead ECG and automated SW-ECG. Measurement Agreement was analyzed using the Bland-Altmann Method. Bias is represented by the solid black line, limit of agreement is represented *via* d dashed red line. Sections of SW-ECG from outliners are shown in red circles. SW-ECG, Single-Lead Withings Electrocardiogram; QTc, corrected QT-interval; AI, Artificial Intelligence.

We enrolled 317 patients (48% female, mean age 63 ± 17 years, Figure 1). Clinical reasons for obtaining an ECG were routine check-up (*n* = 230; 73%), rhythm assessment (*n* = 24; 8%), QT-measurement (*n* = 16; 5%), ischemia (*n* = 4; 1%) and various reasons (*n* = 43, 14%). The AI algorithm measured automatically the following intervals: HR, PR, QRS, and QTc. Among these 317 patients, HR, PR, QRS as well as QTc was automatically calculated by the SW in 295 (93%), 226 (71%), 249 (79%), and 177 patients (56%), respectively. Differences between patients with/without QTc measurements are highlighted in Table 1. Significantly more often the watch was able to detect and measure the QT-interval in younger women without a history of hypertension or heart failure. Reasons for missing automated QT measurement were technical artifacts (noise) such as fibrillation of the baseline or a moving/jumping baseline in 70 patients (50% of all missing), inconclusive recordings (27 cases, 19% of all missing), and tachy- or bradycardia (17 cases, 12% of all missing) amongst others. Manual measures of the QT-interval in the 12-lead ECG were possible in all patients. Two patients were excluded, since there was substantial time difference between ECG recordings. The 175 SW-ECG and 12-lead ECG were recorded within 63 s (95% CI 57–69 s) of each other. Median HR was 69 bpm (interquartile range 62–77). 21% of patients had a heart rate of < 60 or > 100 bpm. When comparing the HR calculated by the SW-AI with the HR manually measured by the cardiologists using the 12-lead ECG, we were able to report a bias of −0.14 bpm with 95% LoA of −8.95 and 7.86 bpm with 6 outliers using the Blant-Altman method. QT-prolonging drugs and/or beta-blocker were present in 70 of 175 patients (40.0%). QTc prolongation defined as ≥ 460 ms for women and ≥ 440 ms for men was noted in 7 (8%) of 91 women and in 10 (12%) of 84 men. AUC of correctly detecting measurements over 460 ms (women) by the SW was 0.91 (95% CI 85–97%) and for measurements over 440 ms (men) 0.89 (95% CI 83–96%). When comparing QTc measurements calculated by the SW-AI with the QTc measurements manually measured by the cardiologists using the 12-lead ECG, we were able to report a bias of 6.6 ms with 95% LoA of −59 and 72 ms using the Blant-Altman method (Figure 2). The disagreement for QTc measurements between the SW-AI and the manual measurements by the cardiologist using the 12-lead ECG was < 15 ms in 38% cases, > 20 ms in 54, and 29% of measurements had a disagreement > 30 ms. In 12 patients (7%) the difference between the QTc-intervals was greater than the LoA. When only including patients with prolonged QTc interval defined as ≥ 460 ms for women and ≥ 440 ms for men, we were able to report a bias of 16 ms with 95% LoA of −78 and 111 ms using the Blant-Altman method. Among the 12 patients (7%) with a greater difference between the QTc-intervals than the LoA, 3 patients (2%) belonged to the subgroup of prolonged QT-intervals. Premature ventricular complexes and noise were observed in most of outliers. Examples are provided in circles in Figure 2. A total of 81 (46%) of patients presented with lower than 20 ms difference between the two QT-measurements in the 12-lead ECG, which is considered perfect (4). Differences between the manual QT-measurements of the cardiologists resulted in a bias of 0.13 ms (95% LoA −15 to 15 ms), measurements in 10 (6%) patients were outside the LoA. In 9 recordings, the remeasurement of a third cardiologist was necessary. A scatterplot showed a linear R2 of 0.94 between measurements of the cardiologists.

**TABLE 1:** Baseline characteristics of patients available for analysis.

	Overall population (*n* = 317)	QT-interval detectable in SW-ECG (*n* = 177)	QT-interval not detectable in SW-ECG (*n* = 140)	
Female sex-no. (%)	151 (48)	93 (56)	58 (41)	*p* < 0.05
Mean age -yr	63.3 (± 17.2)	60.9 (± 18.5)	66.3 (± 14.9)	*p* < 0.01
≥65 yr-no. (%)	171 (54)	85 (48)	86 (61)	*p* < 0.05
BMI, kg/m^2^	26.7 (± 5.2) *n* = 233	26.4 (± 5) *n* = 124	27.0 (± 5.5) *n* = 109	*p* = 0.44
Hypertension-no. (%)	159 (50)	77 (44)	82 (59)	*p* < 0.01
DM-no. (%)	71 (22)	38 (22)	33 (24)	*p* = 0.66
Stroke/TIA-no. (%)	16 (5)	10 (6)	6 (4)	*p* = 0.62
History HF-no. (%)	46 (15)	16 (9)	30 (21)	*p* < 0.01
CAD-no. (%)	87 (27)	42 (24)	45 (32)	*p* = 0.10
Sinus rhythm-no. (%)	242 (90)	165 (93)	119 (85)	*p* < 0.05
AF-no. (%)	33 (10)	12 (7)	21 (15)	*p* < 0.05
**Indication for ECG**				
Ischemia-no. (%)	4 (1)	1 (1)	3 (2)	
Rhythm-no. (%)	24 (8)	13 (7)	11 (8)	
QT-no. (%)	16 (5)	7 (4)	9 (6)	
Routine check-up-no. (%)	230 (73)	125 (71)	105 (75)	
Other -no. (%)	43 (14)	31 (18)	12 (9)	
**Branch block**				
Right BBB-no. (%)	20 (6)	10 (6)	10 (7)	*p* = 0.65
Left BBB-no. (%)	28 (9)	9 (5)	19 (14)	*p* < 0.01
Left AFB-no. (%)	9 (3)	7 (4)	2 (1)	*p* = 0.31

*AF, atrial fibrillation; AFB, anterior fascicular block; BBB, bundle branch block; BMI, body mass index; CAD, coronary artery disease; DM, diabetes mellitus; ECG, electrocardiogram; HF, heart.*

## Discussion

In this clinical validation of a direct-to-consumer smartwatch in a real world cohort of patients we report the following main findings: (1) The automated algorithm was able to measure QTc in 56% of cases. Main reasons for missing QTc measurements were technical artifacts. By manual review, QT-measurements were possible in additional 20% of SW-ECGs. Interestingly, more often the algorithm provided automated measurements in younger women without a history of hypertension or heart failure. It was more challenging for the algorithm to obtain interval measurements in abnormal ECGs, therefore impeding clinical application in patients at risk. In another study (6) using a multilead handheld device, it was shown, that QTc could be obtained by the device most frequent in lead II. Changing of the Scanwatch’s position for recording might lead to more usable data. However, might also be difficult for patients to record. (2) We found fair to good agreement between automated-SW-ECG QTc-measurements and manual 12-lead-QTc measurements. This SW-AI algorithm tends to underestimate the QTc interval which has also been reported with another device (3). Consumers and healthcare providers need to be aware of this discrepancy when applying this technology in real-world patients. (3) Adapting this smartwatch to a clinical setting such as the use of assisted measurement of QT-prolongation screening in pharmacies, we propose that until an improved algorithm is available, manual review is required in a high percentage of recordings to achieve conclusive findings and measurements. However, the SW-AI’s abilities in measuring the heartrate indicate the possibilities of this technology.

Our findings corroborate and extend other studies investigating QTc-intervals through either device based single lead or device assisted multilead-ECG (2–5, 11, 12). This SW is currently the only single-lead open market device offering AI-based easy available QT-measurement. Contrary to the Kardia AliveCor 6L, a six-lead ECG, the Withings SW does not have an FDA-approval for QTc-measurements.

## Limitations

Limitations of this study are as follows: First, although we were able to enroll 317 patients, this is a single center cohort study, we tested a single device on a single position and uniquely lead I was recorded. The comparison between the SW-ECGs lead I measurement and the 12-lead EKGs measurement of lead II could be responsible for the small bias, however, when comparing QTc-intervals measured in lead I from both SW- and 12-lead ECG, findings were confirmed with a bias of 0.7 ms with 95% LOA between −65 and 66 ms with 9 outliers reported. Second, while AI-determined QTc intervals from a single lead ECG can be accurate in monitoring changes in the QTc interval over time, it can never be as accurate for the measurement of the actual QTc when compared to a 12-lead ECG since factors like e.g., QT dispersion can never be accounted for. Third, the time-pressured recording could have contributed to an increased number of tracings of elevated noise possibly leading to a decrease of numbers of successful QT-measurements by the algorithm. No repeat measurements were taken, since they would have increased the time between recordings. This approach, however, allowed us to record the single lead ECG and 12-lead ECG within 63 s (95% CI 57–69 s) of each other. Fourth, despite being the most popular used method for measuring the QTc interval, the tangent method might underestimate the QTc interval as shown by Sharif et al. (13). However, this method was chosen since it allows the most reliable approach and reports a higher reproducibility. Fifth, despite being able to perform measurements within 63 s of each other, the gold standard would have been simultaneously recording SW-ECG and Holter-ECG allowing measurements of QT-intervals without any beat-by-beat variability (14).

## Conclusion

In conclusion, utilization of single-lead SW-ECG for QTc monitoring could be practicable but still needs further validation, algorithm improvement and long-term research to be of possible use in a widespread clinical setting.

## Data Availability Statement

The raw data supporting the conclusions of this article will be made available by the authors, without undue reservation.

## Ethics Statement

The studies involving human participants were reviewed and approved by the Ethikkommission Nordwest- und Zentralschweiz (EKNZ). The patients/participants provided their written informed consent to participate in this study.

## Author Contributions

CV, ML, DM, and EH were involved in data collection and analysis. DM and PB involved in the statistical analysis and wrote the initial manuscript. All authors read, reviewed, and edited the manuscript in the subsequent revision rounds.

## Conflict of Interest

PB received research funding from the “University of Basel“, the “Stiftung für Herzschrittmacher und Elektrophysiologie,” the “Freiwillige Akademische Gesellschaft Basel”, and Johnson & Johnson, all outside the submitted work and reports personal fees from Abbott. SK has received funding of the “Stiftung für Herzschrittmacher und Elektrophysiologie.” CS Member of Medtronic Advisory Board Europe, and Boston Scientitic Advisory Board Europe, received educational grants from Biosense Webster and Biotronik, a research grant from the European Union’s FP7 program and Biosense Webster, and lecture and consulting fees from Abbott, Medtronic, Biosense-Webster, Boston Scientific, Microport, and Biotronik all outside the submitted work. MK reports personal fees from Bayer, personal fees from Böhringer Ingelheim, personal fees from Pfizer BMS, personal fees from Daiichi Sankyo, personal fees from Medtronic, personal fees from Biotronik, personal fees from Boston Scientific, personal fees from Johnson & Johnson, personal fees from Roche, grants from Bayer, grants from Pfizer, grants from Boston Scientific, grants from BMS, grants from Biotronik, and grants from Daiichi Sankyo, all outside the submitted work. BS reports speaker’s bureau for Medtronic. The remaining authors declare that the research was conducted in the absence of any commercial or financial relationships that could be construed as a potential conflict of interest.

## Publisher’s Note

All claims expressed in this article are solely those of the authors and do not necessarily represent those of their affiliated organizations, or those of the publisher, the editors and the reviewers. Any product that may be evaluated in this article, or claim that may be made by its manufacturer, is not guaranteed or endorsed by the publisher.
